# Solution-Processed Cu_2_S Nanostructures for Solar Hydrogen Production

**DOI:** 10.1021/acs.chemmater.2c03489

**Published:** 2023-03-08

**Authors:** Xi Zhang, Stephan Pollitt, Gihun Jung, Wenzhe Niu, Pardis Adams, Jan Bühler, Nora S. Grundmann, Rolf Erni, Maarten Nachtegaal, Neul Ha, Jisu Jung, Byungha Shin, Wooseok Yang, S. David Tilley

**Affiliations:** † Department of Chemistry, University of Zurich, Winterthurerstrasse 190, 8057 Zurich, Switzerland; ‡ Paul Scherrer Institut (PSI), Forschungsstrasse 111, 5232 Villigen, Switzerland; § Department of Materials Science and Engineering, Korea Advanced Institute of Science and Technology (KAIST), 291 Daehak-ro, Yuseong-gu, Daejeon 34141, Republic of Korea; ∥ Electron Microscopy Center, Empa, Swiss Federal Laboratories for Materials Science and Technology, Überlandstrasse 129, 8600 Dübendorf, Switzerland; ⊥ School of Chemical Engineering, Sungkyunkwan University, 2066 Seobu-ro, Jangan-gu, Suwon-si, Gyeonggi-do 16419, Republic of Korea; # SKKU Institute of Energy Science and Technology (SIEST), Sungkyunkwan University, Suwon 16419, Republic of Korea

## Abstract

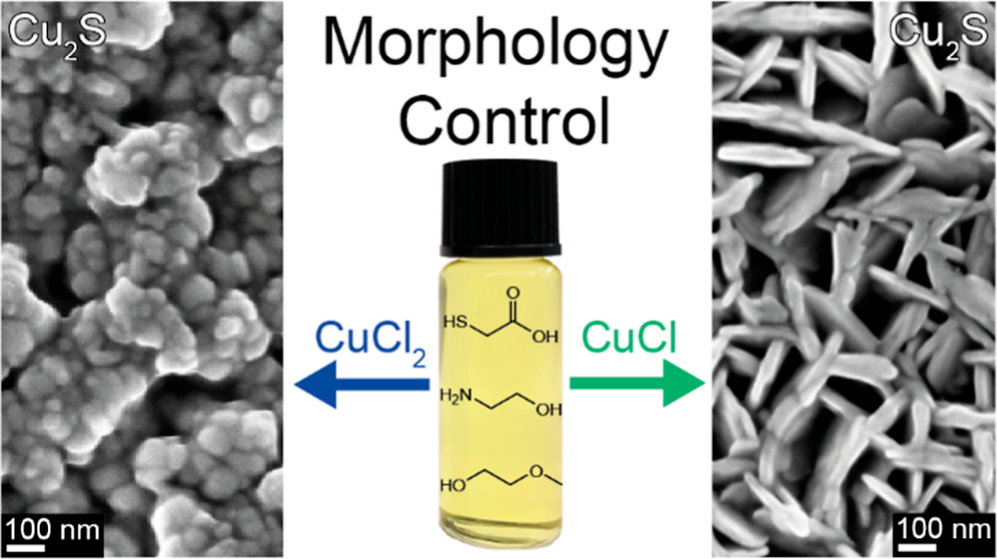

Cu_2_S is a promising solar energy conversion material due to its suitable optical properties, high elemental earth abundance, and nontoxicity. In addition to the challenge of multiple stable secondary phases, the short minority carrier diffusion length poses an obstacle to its practical application. This work addresses the issue by synthesizing nanostructured Cu_2_S thin films, which enables increased charge carrier collection. A simple solution-processing method involving the preparation of CuCl and CuCl_2_ molecular inks in a thiol-amine solvent mixture followed by spin coating and low-temperature annealing was used to obtain phase-pure nanostructured (nanoplate and nanoparticle) Cu_2_S thin films. The photocathode based on the nanoplate Cu_2_S (FTO/Au/Cu_2_S/CdS/TiO_2_/RuO_*x*_) reveals enhanced charge carrier collection and improved photoelectrochemical water-splitting performance compared to the photocathode based on the non-nanostructured Cu_2_S thin film reported previously. A photocurrent density of 3.0 mA cm^–2^ at −0.2 versus a reversible hydrogen electrode (*V*_RHE_) with only 100 nm thickness of a nanoplate Cu_2_S layer and an onset potential of 0.43 *V*_RHE_ were obtained. This work provides a simple, cost-effective, and high-throughput method to prepare phase-pure nanostructured Cu_2_S thin films for scalable solar hydrogen production.

## Introduction

Sunlight is an immense source of clean and sustainable power, providing far more energy than the demand of human society. Although photovoltaics can be used to generate electricity from solar power, the cost-effective storage of solar energy remains an important challenge due to the diurnal cycle and seasonal variability of sunlight. Photoelectrochemical (PEC) water splitting is considered a promising strategy to convert the vast amount of solar energy into storable carbon-neutral hydrogen fuel.^1^ In order to generate hydrogen in a cost-effective manner, the PEC water-splitting system must be efficient and stable, and the fabrication methods have to be cheap and scalable.^2,3^

Photoabsorption and separation of photogenerated charges are crucial efficiency-determining processes in PEC water splitting. These processes are primarily governed by the intrinsic properties of the light-absorbing semiconductor (band gap, light absorption coefficient, minority carrier diffusion length, etc.). However, structural design is also essential in order to boost light harvesting and charge separation.^4^ Nanostructuring strategies have been employed to improve light trapping and charge separation by enhancing light scattering within the photoelectrode and shortening the travel distance of charge carriers, beneficial especially for semiconductors that have a short carrier diffusion length.^5^ To construct particular nanostructures, template-assisted techniques,^6^ vapor–liquid–solid method,^7^ molecular beam epitaxy growth,^8^ and hydrothermal method can be used,^9^ but chemical ink-based solution processing, consisting of the synthesis of inks followed by simple coating and annealing, could be an ideal way owing to its simplicity and effectiveness.

Cu_2_S is one promising p-type semiconductor for solar energy conversion. The advantages of this material are high elemental earth abundance, nontoxicity, and suitable optical properties including an indirect band gap of 1.2 eV, a direct band gap of 1.8 eV, and a high absorption coefficient of over 10^4^ cm^–1^.^10,11^ However, the presence of multiple secondary phases (monoclinic low chalcocite Cu_2_S stable below 105 °C, hexagonal high chalcocite Cu_2_S stable between 105 and 425 °C, cubic γ-chalcocite Cu_2_S stable at temperatures higher than 425 °C, monoclinic djurleite Cu_1.96_S, trigonal digenite Cu_1.8_S, orthorhombic anilite Cu_7_S_4_, and hexagonal covellite CuS) and the short minority carrier diffusion length have posed an obstacle for its practical applications.^12,13^ In our recent study, we reported that phase-pure Cu_2_S thin films can be obtained by thiol–amine-based solution processing for the first time.^14^ Thiol–amine mixtures can be strong solvents, enabling the dissolution of more than 65 bulk inorganic materials, including ZnS which is insoluble in hydrazine, to form molecular inks.^15−19^ Generally, upon a simple mild-temperature annealing step, the solid-state crystalline phase can be recovered. However, the performance of as-prepared Cu_2_S photocathodes was limited, presumably due to the imbalance between light absorption and carrier collection. Even in the literature reports on high-efficiency Cu_2_S/CdS solar cells, nanometer-scale minority carrier diffusion lengths of Cu_2_S,^20−22^ which are incommensurate with the micrometer-scale demand of the penetration depth of solar light,^10,23^ resulted in the loss of photogenerated charge carriers. Thus, the nanostructure design of Cu_2_S is expected to be an effective strategy to overcome the disaccord between the light absorption need and the short charge carrier diffusion length, which can shorten the travel distance of photogenerated carriers, improve the efficiency of charge collection, and therefore enhance the overall efficiency.

In this work, we report a novel strategy to fabricate nanostructured Cu_2_S thin films via solution processing. By simply spin-coating and annealing of molecular inks containing CuCl or CuCl_2_, unique nanostructures of phase pure Cu_2_S were fabricated (nanoplates from the CuCl-based ink and nanoparticles from the CuCl_2_-based ink). Various characterization techniques, including grazing-incidence X-ray diffraction (GIXRD), X-ray photoelectron spectroscopy (XPS), and Raman spectroscopy confirmed that both nanostructures are composed of the pure low-chalcocite Cu_2_S phase, regardless of their morphology. Additionally, local coordination environments and molecular structures of both inks were analyzed by X-ray absorption spectroscopy (XAS) and liquid-phase Raman spectroscopy to determine the link between ink composition and film morphology. The photocathode based on the nanoplate Cu_2_S (FTO/Au/Cu_2_S/CdS/TiO_2_/RuO_*x*_) revealed enhanced performance compared to the non-nanostructured counterpart, benefiting from the advantages of the nanostructure. We believe this simple method to prepare novel Cu_2_S nanostructures reported here will be the first step toward revisiting Cu_2_S semiconductor as a promising light absorber for solar energy conversion.

## Experimental Section

### Materials

Copper(I) chloride beads (CuCl, anhydrous, ≥99.99%, Sigma-Aldrich), copper(II) chloride powder (CuCl_2_, anhydrous, ≥99.995%, Sigma-Aldrich), copper(I) oxide powder (Cu_2_O, anhydrous, ≥99.99%, Sigma-Aldrich), copper(II) oxide powder (CuO, 99.999%, Sigma-Aldrich), ammonium chloride powder (NH_4_Cl, anhydrous, ≥99.5%, Sigma-Aldrich), 2-methoxyethanol (2ME, anhydrous, 99.8%, Sigma-Aldrich), ethanolamine (EA, ≥99.5%, Sigma-Aldrich), thioglycolic acid (TGA, ≥99%, Sigma-Aldrich), cadmium sulfate (CdSO_4_, ≥99.99%, Sigma-Aldrich), ammonia solution (NH_3_·H_2_O, 25%, Honeywell), thiourea (≥99.0%, Sigma-Aldrich), potassium perruthenate (KRuO_4_, 97%, Alfa Aesar), and hydrophobic polytetrafluoroethylene (PTFE) filters (0.20 μm pore size, 13 mm diameter, Merck) were used as received.

### Preparation of Molecular Inks

In the consideration of good wettability on substrates and high quality of the resulting thin films, 0.35 M CuCl ink, 0.35 M CuCl_2_ ink, 0.175 M Cu_2_O ink, 0.35 M CuO ink, and (0.35 M CuCl + 0.35 M NH_4_Cl) ink were prepared by separately dissolving CuCl beads (0.142 g), CuCl_2_ powder (0.193 g), Cu_2_O powder (0.103 g), CuO powder (0.114 g), and CuCl beads (0.142 g) together with NH_4_Cl powder (0.077 g) in a solvent mixture of 2ME (2.5 mL), EA (1.0 mL), and TGA (0.6 mL) with stirring overnight at room temperature in a N_2_-filled glovebox. The volumetric ratio between EA and TGA was chosen based on the literature.^16^ The inks were stable for months if closed tightly free from water and oxygen.

### Preparation of Cu_2_S Thin Films

100 nm Au (with 10 nm Cr adhesion layer) was first coated on 2.5 × 2.5 cm FTO substrates by thermal evaporation at room temperature using an Oxford Vacuum Science VapourPhase/PicoSphere system. Prior to spin coating, the quartz, FTO, and Au/FTO substrates were treated by a UV–ozone cleaner for 15 min, and 0.35 M CuCl and CuCl_2_ inks were filtered by PTFE filters to remove any undissolved aggregations or impurities. A static dispense spin-coating process was applied using a Laurell Technologies Corporation WS-650Mz-23NPPB single-wafer spin processor by uniformly spreading ∼0.15 mL ink onto the entire quartz, FTO, or Au/FTO substrates and then spin coating at 2500 r.p.m. for 30 s with an acceleration rate time of 1 s in a N_2_-filled glovebox. In between coatings, the films were quickly dried on a hot plate (also in the N_2_-filled glovebox) at 350 °C for 2 min and then allowed to cool to room temperature before the next coating. After (typically) four coats, a final annealing process at 350 °C for 30 min was performed.

### Chemical Bath Deposition of CdS Layer

The setup for the chemical bath deposition (CBD) of the CdS layer consisted of a jacketed beaker (1 L internal dimension) and a Thermo Scientific Arctic Series refrigerated/heated bath circulator (SC150 immersion circulator, A25 stainless steel bath). First, the Cd solution was prepared by dissolving CdSO_4_ (126.6 mg, 0.61 mmol) and NH_3_^.^H_2_O (41.66 mL, 1.07 mol) in deionized (DI) water (260 mL) in the jacketed beaker. The bath circulator was turned on and stirred at 300 r.p.m. and was allowed to warm up for 30 min until the temperature of the bath reached and remained constant at 60 °C. The thiourea solution was then prepared by dissolving thiourea (2.85 g, 37.4 mmol) into 33.33 mL of DI water and added to the beaker, forming the final CdS deposition solution of 1.82 mM CdSO_4_, 0.11 M thiourea, and 3.19 M NH_3_·H_2_O, which was stirred for 3 min to get a stable CdS deposition rate. Cu_2_S thin films were then immersed into the solution for 4 min. After the CdS deposition, the samples were rinsed with DI water, dried under a stream of N_2_, and then immediately put into the atomic layer deposition (ALD) reactor for the subsequent deposition of the TiO_2_ layer.

### ALD of TiO_2_ Layer

The TiO_2_ layer was deposited by ALD using a Picosun R200 system. Tetrakis(dimethylamido)titanium (TDMAT) and H_2_O were used as the Ti and O sources, respectively. The temperature of the TDMAT precursor cylinder was held at 85 °C, and the reactor temperature was 120 °C. A total of 1860 ALD cycles were carried out to enable the thickness of the TiO_2_ layer of ∼100 nm, which was confirmed by ellipsometry on a silicon witness wafer.

### Photoelectrodeposition of RuO_*x*_ Catalyst

1.3 mM KRuO_4_ solution was freshly prepared by dissolving KRuO_4_ (6 mg) in DI H_2_O (20 mL) before each deposition. Typically, the catalyst was deposited at a constant current density of −28.3 μA cm^–2^ for 15 min under simulated one sun illumination.^24^

### Characterization of Cu_2_S Thin Films

The crystal structures of prepared Cu_2_S thin films were examined by X-ray diffraction using a Rigaku Smartlab diffractometer at 2° min^–1^ with a step width of 0.01° in the grazing incidence mode to avoid strong diffraction peaks from the Au/FTO substrate. XPS was performed using a physical electronics (PHI) Quantum 2000 X-ray photoelectron spectrometer featuring monochromatic Al Kα radiation, generated from an electron beam operated at 15 kV and 32.3 W. The energy scale of the instrument was calibrated using an Au reference sample. The analysis was conducted at 1 × 10^–6^ Pa, with an electron take-off angle of 45° and a pass energy of 46.95 eV. Core-level binding energies were determined by fitting Voigt profiles (GL80) after Shirley background subtraction. Charge neutralization was used throughout the measurement. The Raman spectra were obtained using a Renishaw System at an excitation wavelength of 532 nm with a laser spot size of around 2 μm. The microstructures were characterized with scanning electron microscopy (SEM, Zeiss Gemini 450) and transmission electron microscopy (TEM, Hitachi HT7700 EXALENS). High-angle annular dark-field scanning TEM (HAADF–STEM) images and STEM–energy-dispersive X-ray spectroscopy (EDX) elemental maps were obtained with an FEI Titan Themis microscope equipped with a hexapole-type aberration corrector for STEM (CEOS DCOR) and a Super-X EDX system, operated at 300 kV. The transmittance and reflectance spectra of Cu_2_S thin films were measured by UV–visible spectroscopy (Shimadzu UV 3600 Plus).

### Characterization of Molecular Inks

0.35 M CuCl and CuCl_2_ inks placed in quartz capillaries with a diameter of 1.5 mm under an inert gas atmosphere and sealed by wax were used for XAS measurements. Cu K-edge XAS was performed at the SuperXAS beamline of the Swiss light source (SLS), which operates in the top-up mode at 2.4 GeV and a ring current of 400 mA. The polychromatic X-rays from a 2.9 T superbend magnet were collimated by a silicon-coated mirror (which also served to reduce higher-order harmonics) and subsequently monochromatized by a Si(111) channel-cut crystal. The monochromator was operated at a frequency of 1 Hz resulting in two spectra per second. The beam was focused by a Rh-coated toroidal mirror to a spot size of 600 × 200 (*H* × *V*) micrometer at the sample position. The Cu K-edge X-ray absorption spectra were collected in the transmission mode using 15 cm long ionization chambers filled with 1 bar of N_2_ and by measuring a Cu foil simultaneously for absolute energy calibration. For each Cu ink sample, 239 quick XAS spectra were taken over the course of 2 min and subsequently averaged. Spectra of the reference compounds were taken from pellets of the corresponding powders and collected in the same way as the inks. Background subtraction, normalization, interpolation, and averaging were done with the python-based software ProQEXAFS.^25^ X-ray absorption near-edge structure (XANES) analysis and fitting of the extended X-ray absorption fine structure (EXAFS) region were performed with Larch.^26^ Crystal structures of Chalcocite (Cu_2_S) and Nantokite (CuCl) served as models for the first shell fits.^27,28^ The intrinsic loss factor S_0_^2^ was determined by the fitting of a copper foil reference and was fixed to 0.8. Mean square disorder σ^2^ and coordination number CN were constrained to positive values. *E*_0_ remained unconstrained. Fitting was performed in the *R*-space from 0.9 to 2.9 Å, which was obtained by Fourier transforming the EXAFS spectra from 3 to 12 Å^–1^. The liquid-phase Raman spectra were obtained using a Renishaw System at an excitation wavelength of 532 nm with a laser spot size of around 2 μm.

### PEC Characterization of Cu_2_S Photocathodes

PEC performance of the Cu_2_S photocathodes was carried out in a three-electrode electrochemical cell. A two-channel potentiostat (BioLogic SP-300) was used to control the potential of the working electrode. A Pt wire and an Ag/AgCl electrode (KOSLOW, saturated KCl, +0.197 V vs normal hydrogen electrode) were used as counter and reference electrodes, respectively. The electrolyte used in all PEC measurements was 1 M phosphate buffer (K_2_HPO_4_/KH_2_PO_4_, pH 7). The light source was a 150 W Xe-lamp (LOT Oriel) equipped with an AM 1.5 G filter, and the intensity (100 mW cm^–2^) was calibrated with a standardized silicon solar cell (PV Measurements, USA). Incident photon-to-current efficiency (IPCE) was measured in a home-built system equipped with a halogen light source and a double monochromator. The light intensity was measured with a calibrated silicon photodiode before each measurement. The absorbed photon-to-current efficiency (APCE) is calculated by eq 1.^29,30^
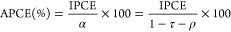
1where α is the absorptance, τ is the transmittance, and ρ is the reflectance of Cu_2_S thin films prepared on FTO substrates. The stability test of the Cu_2_S photocathode based on the nanoplate Cu_2_S thin film was measured at 0 *V*_RHE_ in the same setup as that for PEC measurement.

### Gas Product Analysis

The gas product was generated in an airtight three-finger cell and analyzed by gas chromatography (GC, Bruker GC-450) with a 3 × 2 mm packed molecular sieve 13 × 80–100 column. A constant stream of H_2_O-saturated Ar (6.0 L min^–1^) went through the sample as the carrier gas followed by a drying tube and connected to the gas chromatograph to allow for frequent analysis of the composition of the gas product. The column and reference gas (Ar) flow were set at 20 mL min^–1^. The oven was operated isothermally at 100 °C, and the gas product was detected by a thermal conductivity detector. The retention time is about 1 min for H_2_. The detection limit of this setup is 0.3 pmol H_2_ s^–1^. Before each measurement, the whole system was purged with Ar for 30 min, and the removal of residual gases such as oxygen and nitrogen was confirmed by sampling the headspace of the airtight cell. Calibration of the hydrogen peak area was carried out in a two-electrode system using two Pt wires as the working electrode and the counter electrode and obtained by GC. Chronopotentiometry method was adopted to apply a constant current between two Pt wires, and in 30 min, the saturated hydrogen peak area was matched with an applied current assuming 100% Faradaic efficiency for calibration. Three calibration points were made by applying different currents (0.1, 0.5, and 1.0 mA), and then the obtained linear calibration curve were used to convert current into the hydrogen peak area. To measure the H_2_ evolution from the Cu_2_S photocathode, the same three-electrode system as the PEC measurement was set, a light-emitting diode (LED) white lamp was used as the light source, and a potential of 0 *V*_RHE_ was applied. Current acquired under the LED lamp was matched to the current under a simulated AM 1.5 G by adjusting the LED lamp power. The evolved gas was collected into the gas-sampling loop of the gas chromatograph every 5 min. The photocurrent of the Cu_2_S photocathode was integrated every 5 min and converted into the hydrogen peak area by using a calibration curve. Faradaic efficiency was calculated by dividing the detected hydrogen peak area by the calculated hydrogen peak area from the photocurrent.

## Results and Discussion

### Synthesis and Characterization of Nanostructured Cu_2_S Thin Films

**Figure 1:**
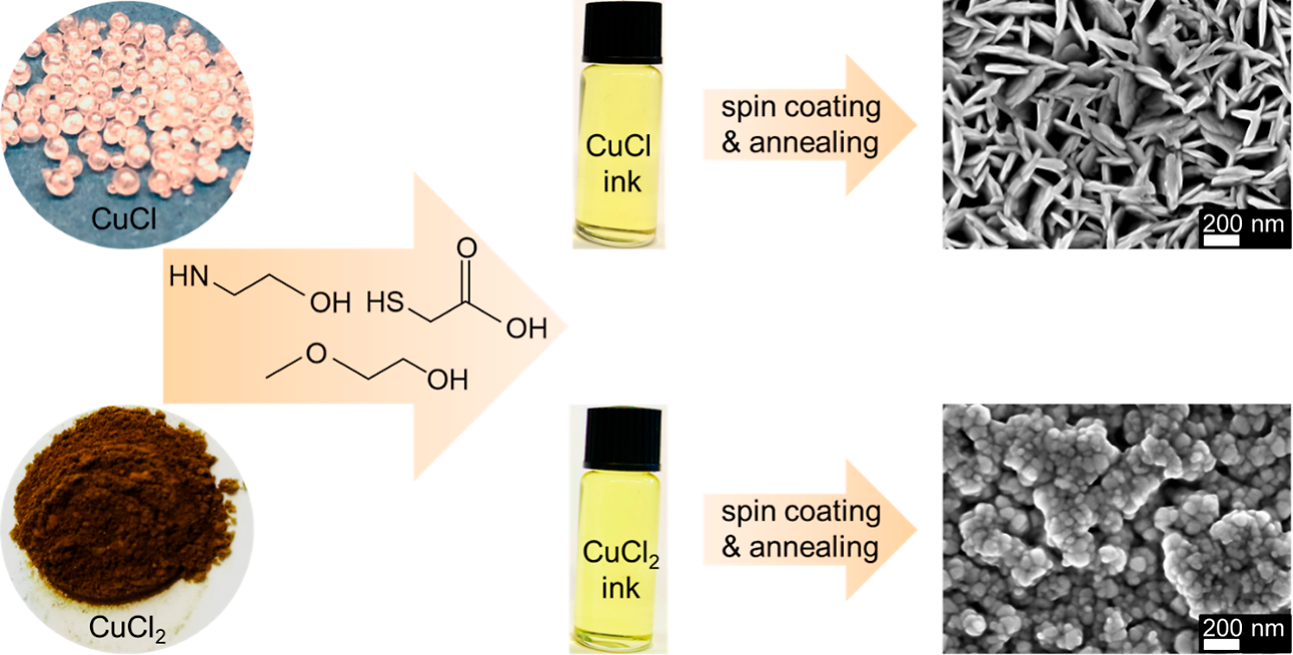
Schematic diagram showing the preparation process of the molecular inks, and plain-view SEM images of the resulting thin films prepared from the 0.35 M CuCl ink and the 0.35 M CuCl_2_ ink.

Anhydrous CuCl and CuCl_2_ precursors were dissolved in a solvent mixture of 2ME, EA, and TGA to a final concentration of 0.35 M, yielding clear, sedimentation-free molecular inks (Figure 1). By simply spin coating the inks on Au/FTO substrates and annealing at 350 °C for 30 min, different nanostructured thin films were formed. The CuCl-based ink renders a two-dimensional nanoplate morphology perpendicularly oriented to the substrate (Figure S1). The diameter of each plate is around 200 nm, which corresponds to the thickness of the entire film. In contrast, a very different morphology was observed for the thin films derived from the similarly colored CuCl_2_ ink, which consists of small nanoparticles with diameters of ∼50 nm.

**Figure 2:**
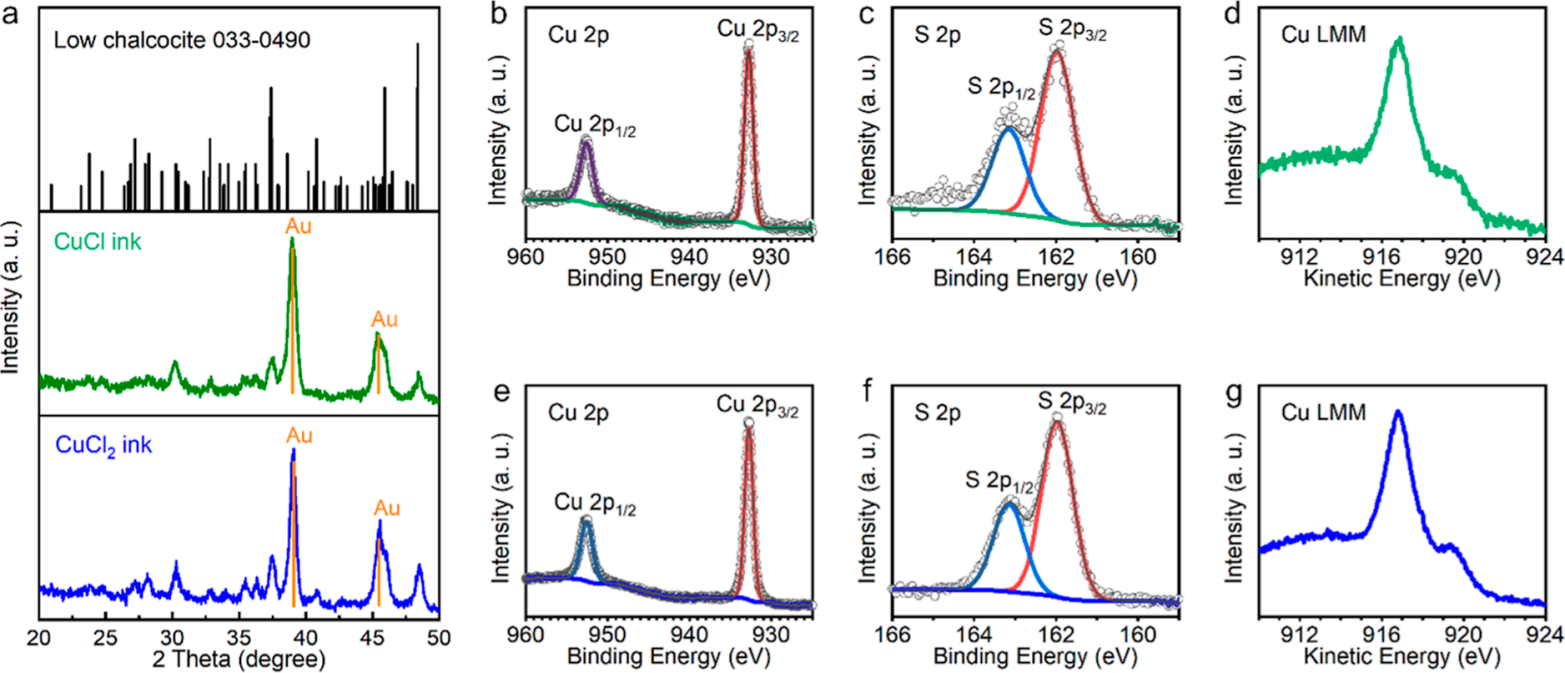
(a) GIXRD patterns of the thin films prepared from 0.35 M CuCl and CuCl_2_ inks. (b–d) Cu 2p XPS spectrum, S 2p XPS spectrum, and Cu LMM Auger spectrum of the thin film prepared from 0.35 M CuCl ink. (e–g) Cu 2p XPS spectrum, S 2p XPS spectrum, and Cu LMM Auger spectrum of the thin film prepared from 0.35 M CuCl_2_ ink.

Phase purities of the nanostructured thin films were examined by GIXRD, XPS, and Raman. GIXRD was performed to reduce the intensity of the Au substrate peaks and to maximize the peak intensities from the nanostructured thin films. As shown in Figure 2a, almost identical GIXRD patterns were observed from both nanoplate and nanoparticle thin films. All diffraction peaks including three diagnostic peaks at 37.4, 45.9, and 48.4° match well with the monoclinic low chalcocite Cu_2_S reference (JCPDS 033-0490), without impurity peaks from CuS, Cu_1.8_S, or Cu_1.96_S phases. The GIXRD results indicate that both the films primarily consist of chalcocite Cu_2_S crystals, even in the film derived by the CuCl_2_ ink, where Cu^2+^ was incorporated as the precursor. XPS was further utilized to verify the composition and phase purity of the nanostructured thin films. In agreement with the GIXRD data, the XPS spectra of the nanoplate and nanoparticle thin films are quite similar. The doublet peaks at 932.8 and 952.6 eV corresponding to Cu 2p_3/2_ and Cu 2p_1/2_ core levels (Figure 2b,e) and the doublet peaks at 162.0 and 163.2 eV corresponding to S 2p_3/2_ and S 2p_1/2_ core levels (Figure 2c,f) match well with the reference XPS values of Cu^+^ and S^2–^ in Cu_2_S.^11,31,32^ It is worth noting that no satellite peaks around 965 eV or between 940 and 945 eV were found in both XPS spectra of Cu 2p orbitals, revealing the absence of Cu^2+^ in both thin films. The broad signal around 165 eV in the S 2p XPS spectrum of the nanoplate thin film (Figure 2c) could not be fit to disulfides with a binding energy between 163.5 and 164 eV or oxidized sulfur species such as sulfonate with a binding energy higher than 166 eV and may be due to a small amount of thiol molecules (−SH) on the sample surface.^33^ Cu LMM Auger spectra (Figure 2d,g) from both nanostructured thin films show a main peak at the kinetic energy of 916.8 eV and a shoulder around 920.0 eV without components at 918.6 eV (which would represent Cu metal), in agreement with the literature XPS results of Cu_2_S free from metallic Cu.^31^ In the XPS spectra of the O 1s orbitals, a single peak at 532.2 eV was observed in both nanostructured samples, far from metal-oxide lattice signals that usually lie between 529 and 530 eV, and is assigned to surface hydroxyl (−OH) groups (Figure S2a,c).^32,34^ Notably, no Cl 2p orbital signals were observed in the XPS spectra (Figure S2b,d). Raman spectroscopy was further performed on the nanostructured thin films. No discernible peaks were observed between 200 and 800 nm in the Raman spectra of both nanoplate and nanoparticle thin films (Figure S3). The absence of a disulfide (−S–S−) stretching signal at 472 cm^–1^ (which can be observed in the CuS phase) agrees with the XPS data and confirms that the broad signal in the S 2p XPS spectrum of the nanoplate thin film is not a disulfide signal.

**Figure 3:**
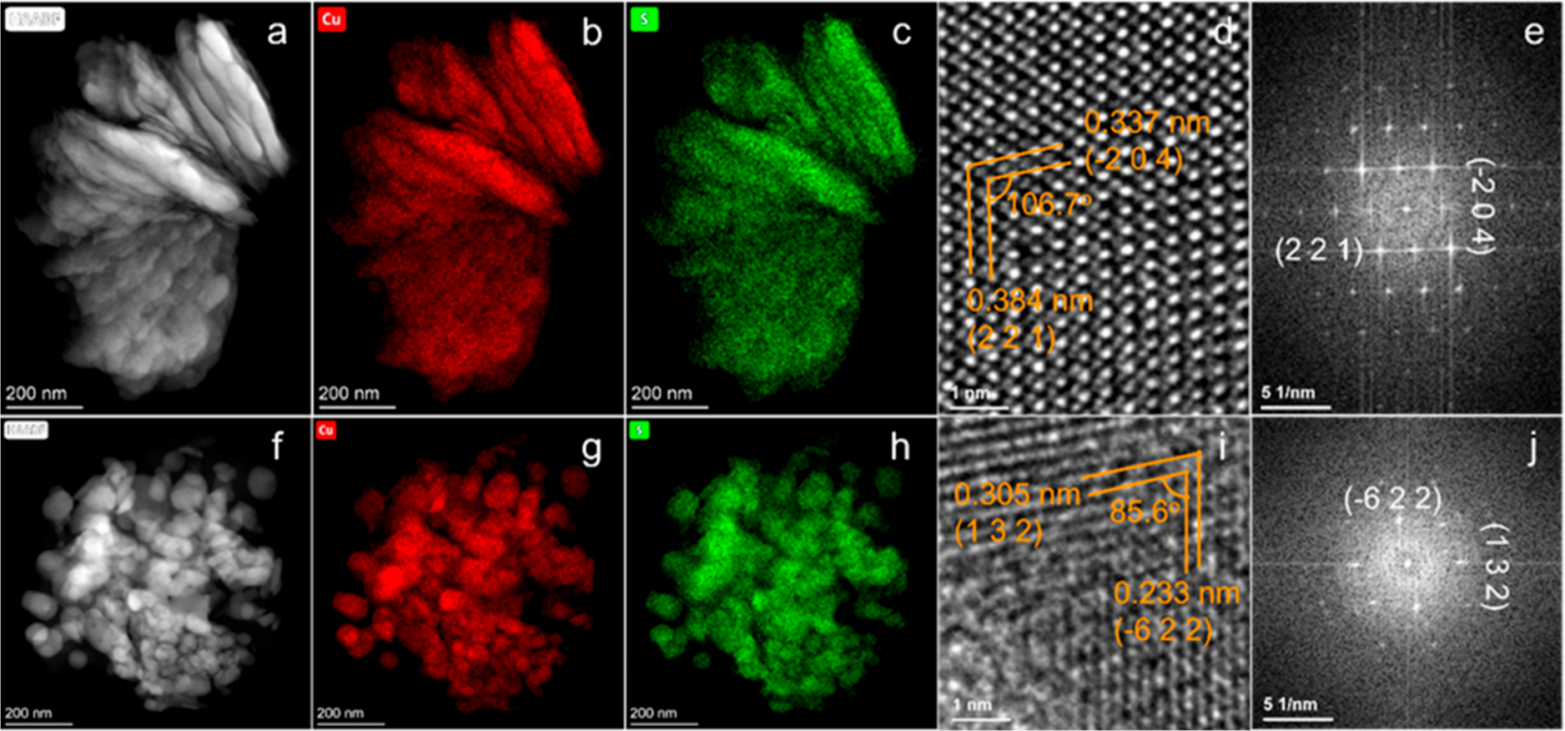
(a,f) HAADF–STEM images and the corresponding STEM–EDX elemental mapping of Cu (b,g) and S (c,h) of nanoplates (a–c) and nanoparticles (f–h) scratched from prepared thin films. (d) High-resolution TEM image taken from (a), and the (e) corresponding FFT image. (i) High-resolution TEM image taken from (f), and the (j) corresponding FFT pattern.

HAADF–STEM and EDX spectroscopy were carried out to further determine the crystal structure and composition of the nanostructured thin films. Representative HAADF–STEM images and the corresponding STEM–EDX elemental mapping images of nanoplates and nanoparticles are shown in Figure 3, revealing uniform distributions of Cu and S throughout the nanoplates and nanoparticles. Additionally, no indication of interfacial breaks from coating iterations was observed. A high-resolution TEM image of the nanoplates is shown in Figure 3d. The interplanar *d*-spacings of 0.337 and 0.384 nm for planes at an angle of 106.7° can be indexed to the (−2 0 4) and (2 2 1) planes of low chalcocite Cu_2_S with a monoclinic crystal structure, corresponding to the fast Fourier transform (FFT) pattern in Figure 3e. For the nanoparticles, the interplanar *d*-spacings of 0.305 and 0.233 nm for planes at an angle of 85.6° could be indexed to the (1 3 2) and (−6 2 2) planes of monoclinic low chalcocite Cu_2_S (Figure 3i–j). In brief, characterizations of the nanoplate and nanoparticle thin films show that both films are phase-pure Cu_2_S despite the different oxidation states of Cu in the precursors, and the morphology of the Cu_2_S is the only clear difference between the two films.

Optical characterization was carried out by measuring the reflectance of nanostructured Cu_2_S thin films prepared on quartz substrates (Figure S4), and the Tauc method was utilized to determine their band gap energy from the reflectance spectra.^35^ As shown in Figure S5, nanoplate Cu_2_S displays an indirect band gap of 1.14 eV and a direct band gap of 1.28 eV, while nanoparticle Cu_2_S shows an indirect band gap of 1.08 eV and a direct band gap of 1.47 eV.

### Copper Chloride Dissolution and Nanostructured Thin-Film Formation Mechanism

The preparation process of nanostructured Cu_2_S thin films is fascinating in that the same TGA_EA_2ME solvent mixture was used to dissolve two kinds of anhydrous copper chlorides with different oxidation states of copper, and then after spin coating and annealing of those two molecular inks, different nanostructure morphologies of Cu_2_S thin films were obtained. To understand the reasons or mechanisms why different nanostructured Cu_2_S were formed in the process might be of great significance for future studies to prepare nanostructured materials via a simple solution-processing approach.

**Figure 4:**
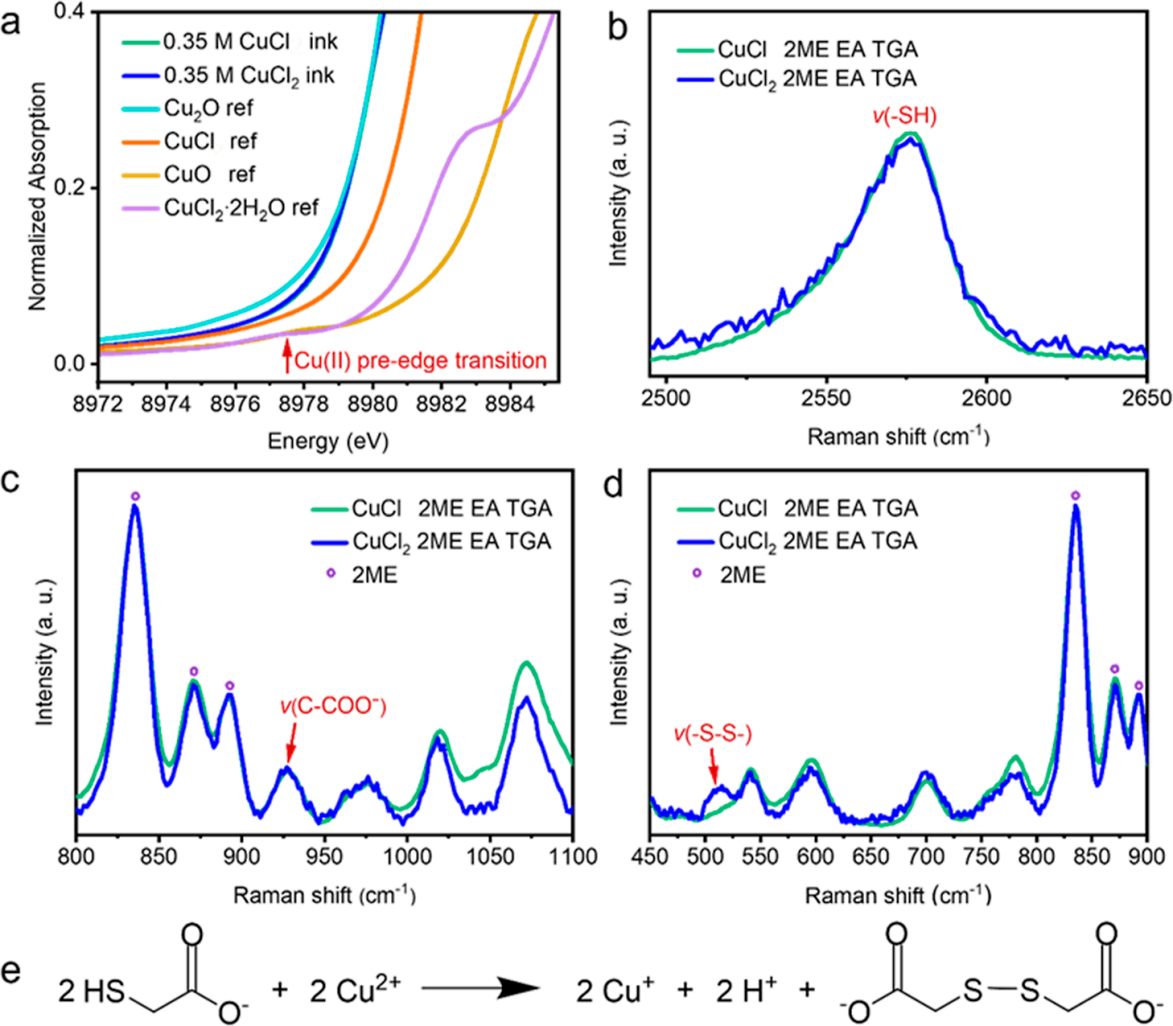
(a) Pre-edge region of the normalized Cu K-edge XANES spectra for 0.35 M CuCl and CuCl_2_ molecular inks (which overlap) along with pressed Cu_2_O, CuCl, CuO, and CuCl_2_·2H_2_O powder references. (b–d) Molecular structure analysis of 0.35 M CuCl and CuCl_2_ molecular inks by liquid-phase Raman spectroscopy normalized according to the 2ME peaks. (e) Reaction between thioglycolate and Cu^2+^ species with the formation of Cu^+^, H^+^, and dithiodiglycolate.

XAS measurements were performed at the Cu K-edge in order to gain insight into the oxidation state and local coordination environment of copper in the 0.35 M CuCl and CuCl_2_ molecular inks in the TGA_EA_2ME solvent mixture. The collected XANES spectra are shown in Figure S6, while the pre-edge region of the normalized XANES spectra is displayed in Figure 4a. XANES spectra of CuCl and CuCl_2_ molecular inks overlap fully, suggesting the formation of products with the same oxidation state of Cu and also the same local environment surrounding Cu. Powder Cu_2_O, CuCl, CuO, and CuCl_2_·2H_2_O were used as references. The white line position is similar to Cu_2_O and is clearly different from CuO and CuCl_2_·H_2_O. Furthermore, in Figure 4a, a characteristic pre-edge signal at around 8977.5 eV is present in the CuO and CuCl_2_·2H_2_O references for Cu(II) but not visible in the Cu_2_O and CuCl references for Cu(I). This low-intensity pre-edge feature is due to a dipole–forbidden transition of a 1s core electron of copper(II) to a 3d orbital, while it is not observed for Cu(I) species because the transition of a 1s electron to the fully filled 3d orbitals of Cu(I) is not possible. The absorption edges of 0.35 M CuCl and CuCl_2_ molecular inks both appear at 8980.0 eV without pre-edge transition signals, indicating that Cu^2+^ was reduced to Cu^+^ in the CuCl_2_ molecular ink. Therefore, the oxidation state of copper is Cu^+^ in both CuCl- and CuCl_2_-based molecular inks.

In order to obtain information on the local environment of Cu, EXAFS spectroscopy was performed. As shown in Figure S7, the Fourier-transformed EXAFS spectra revealed next nearest neighbors with a single bond distance of about 1.8 Å (not phase corrected), which is typical for Cu–Cl and Cu–S–R.^27,28^ Bond distances related to oxygen and nitrogen could not be observed and can be excluded as binding partners for this reason.^36−38^ Fitting results based on chalcocite (Cu_2_S) and nantokite (CuCl) are listed in Table S1 and displayed in Figure S7, giving reasonable results for both Cl and S as binding partners,^27,28^ which cannot be differentiated due to their similar scattering. However, in both inks, Cu forms a complex with either two- or threefold coordination to Cl and S. The XAS data indicate that both inks have identical local coordination environment of copper, inclusive of the oxidation state, bond distance, and coordination number, ruling out that the morphological difference observed originated from the difference in the local coordination around the copper ion in the inks.

Liquid-phase Raman spectroscopy was further employed to detect the molecular structure of 0.35 M CuCl and CuCl_2_ molecular inks. The peak observed at 2573 cm^–1^ in Figure 4b could be assigned to the −SH stretching mode. The peak at ∼930 cm^–1^ in Figure 4c is ascribed to C–COO^–^ stretching, while no peak at ∼907 cm^–1^ corresponding to C–COOH stretching was observed.^39^ These results indicate that the amine group in EA deprotonated mainly the carboxylic acid group rather than the thiol group in TGA to form carboxylate anions, owing to the lower p*K*_a_ value of the carboxylic acid group (p*K*_a_ = 3.83) compared to the thiol group (p*K*_a_ = 9.3).^40^ A major difference was found in the low wavenumber range in Figure 4d, where a broad peak between 500 and 525 cm^–1^ appears only in the 0.35 M CuCl_2_ molecular ink. This signal is characteristic of a −S–S– bond^41^ and could be formed by the oxidation of the thiol group in TGA,^42^ the reaction of which is displayed in Figure 4e. The reduction of Cu^2+^ to Cu^+^ in the CuCl_2_ molecular ink therefore coincides with the oxidation of the thiol group.

Similar phenomena were observed when similar concentrations of CuCl and CuCl_2_ were dissolved separately in a 1:1 molar mixture of 1-propanethiol and *n*-butylamine in the work by Agrawal et al., in which the mass spectrum and X-ray absorption spectra of the two solutions were essentially identical apart from the presence of disulfide in the CuCl_2_ solution, solely identified by Raman spectroscopy as an oxidation product when copper(II) was reduced to copper(I).^43^ The copper complexes detected by mass spectrometry included copper thiolates, copper chlorides, and copper thiolate chlorides without copper-amine complexes.^43^

Based on the discussion above, one major difference between the CuCl and CuCl_2_ inks is the presence of the disulfide bond in the CuCl_2_ ink. Another distinction is the concentration of chloride ions, with the CuCl_2_ ink containing twice as much chloride as the CuCl ink. In order to deconvolute the effects of those two parameters on the final film morphologies, two control experiments were conducted. The first control experiment was the preparation of thin films from 0.175 M Cu_2_O ink and 0.35 M CuO ink in the same TGA_EA_2ME solvent mixture. Figure S8a,b displays the photographs of 0.175 M Cu_2_O ink and 0.35 M CuO ink, which are both clear, homogenous, and yellow-colored solutions. The only difference in the corresponding copper-oxide inks compared to the copper-chloride inks is the absence of chloride ions and the presence of water. The Raman disulfide peak is also observed in the CuO ink (Figure S8c). After spin coating and annealing of the 0.175 M Cu_2_O and 0.35 M CuO inks on Au/FTO substrates under the same conditions, the phase purity and microstructure of the resulting thin films were examined by GIXRD and SEM. Figure S8d shows that the GIXRD patterns of both thin films are chalcocite Cu_2_S but with one or two unknown impurity peaks (not belonging to copper oxides nor copper chlorides). More importantly, a similar microstructure in the form of nanoparticles was observed in the plain-view SEM images (Figure S8e,f), indicating that the presence of the disulfide bond in the ink has no direct influence on the morphology transition from nanoparticles to nanoplates and that the presence of chloride ions is essential for the formation of the nanoplate structure. By comparing the SEM images of thin films synthesized from the Cu_2_O, CuO, and CuCl_2_ inks, it is also apparent that the presence of both chloride ions and the disulfide bond plays a role in the size of the as-prepared nanoparticles. The second control experiment was the preparation of thin films from (0.35 M CuCl + 0.35 M NH_4_Cl) ink to see the effect of absolute concentration of chloride ions. As shown in Figure S9, the (0.35 M CuCl + 0.35 M NH_4_Cl) ink in which the Cl concentration is equivalent to that in the CuCl_2_ ink-is clear yellow, and the resulting thin film has the nanoplate structure. These experiments suggest that it is the combination of chloride ions and disulfide bond in the ink that affects the final morphology of the prepared thin films rather than the absolute concentration of chloride ions.

### PEC Water Splitting Performance

**Figure 5:**
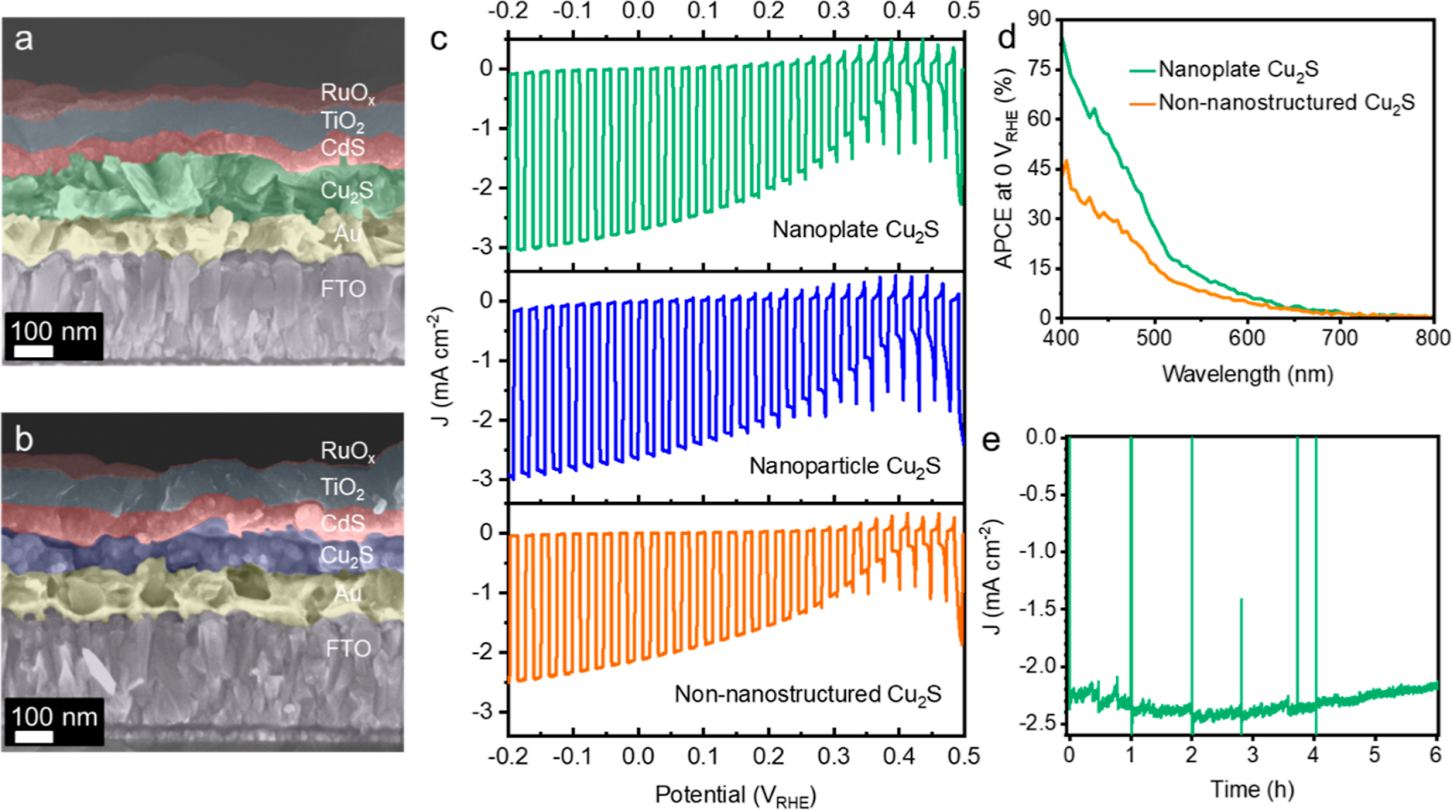
(a) Cross-sectional SEM image of the Cu_2_S photocathode (FTO/Au/Cu_2_S/CdS/TiO_2_/RuO_*x*_) based on the nanoplate Cu_2_S thin film. (b) Cross-sectional SEM image of the Cu_2_S photocathode (FTO/Au/Cu_2_S/CdS/TiO_2_/RuO_*x*_) based on the nanoparticle Cu_2_S thin film. (c) *J*–*E* curves of Cu_2_S photocathodes based on different structures of Cu_2_S thin films under simulated on–off AM1.5 G illumination (100 mW cm^–2^). (d) Comparison of the APCEs of Cu_2_S photocathodes based on the nanoplate Cu_2_S thin film and the non-nanostructured Cu_2_S thin film. (e) Chronoamperometry of the Cu_2_S photocathode based on the nanoplate Cu_2_S thin film under constant bias at 0 V_RHE_ under simulated AM1.5 G illumination (100 mW cm^–2^). All measurements were performed in a 1.0 M phosphate buffer solution (pH 7.0).

The PEC performance of both Cu_2_S nanoplate and nanoparticle thin films were tested in a pH 7 electrolyte with FTO/Au/Cu_2_S/CdS/TiO_2_/RuO_*x*_ device configurations, where 50 nm CdS was deposited on top of Cu_2_S thin films by CBD as the n-type junction layer, 100 nm TiO_2_ was deposited onto the CdS layer by ALD as the protective layer, and 40 nm of photoelectrodeposited RuO_*x*_ served as the hydrogen evolution reaction catalyst to facilitate the charge transfer from the electrode to the electrolyte (Figure 5a,b).^24^ Cu_2_S thin films prepared by a similar thiol–amine-based solution-processing method were considered as the non-nanostructured Cu_2_S ref (14). Figure 5c shows the current density response upon on–off simulated solar irradiation, and the onset potential could be determined by cyclic voltammetry (CV) scans in Figure S10. The reference photocathode based on the non-nanostructured Cu_2_S thin film gave a photocurrent density of 2.5 mA cm^–2^ at −0.2 *V*_RHE_ with an onset potential of 0.42 *V*_RHE_, where the thickness of the Cu_2_S layer was 220 nm.^14^ Even though the thickness of the nanoplate Cu_2_S layer in the photocathode is around 100 nm, the photocathode based on the nanoplate Cu_2_S thin film gave a photocurrent density of 3.0 mA cm^–2^ at −0.2 *V*_RHE_ with a similar onset potential of 0.43 *V*_RHE_. The photocathode based on the same 100 nm of the nanoparticle Cu_2_S thin film gave a slightly lower photocurrent density (2.7 mA cm^–2^ at −0.2 *V*_RHE_) but still superior to the photocathode based on the non-nanostructured Cu_2_S thin film with double the thickness. The onset potential of the photocathode based on the nanoparticle Cu_2_S thin film at 0.45 *V*_RHE_ is around the same range as the other two photocathodes. The PEC performance depending on the thickness of nanostructured Cu_2_S thin films was also tested under the same experimental conditions. As displayed in Figure S11, when the thickness of the nanostructured Cu_2_S thin films exceeded 100 nm, the photocurrent density of the as-prepared photocathodes was reduced, which is probably due to the recombination of photoexcited electrons and holes because of the short minority carrier diffusion length of Cu_2_S. In addition, though the microstructure of the RuO_*x*_ layer was affected by the different nanostructures of Cu_2_S thin films underneath (Figures 5a,b and S11), the nanoplate structure was not 100% maintained in the RuO_*x*_ layer. Therefore, in order to achieve competitive performance with the state-of-the-art Cu_2_S photocathode (FTO/Au/Cu_2_S/CdS/TiO_2_/RuO_*x*_), showing a 6.8 mA cm^–2^ photocurrent at −0.2 *V*_RHE_ and an onset potential of 0.48 *V*_RHE_, with the Cu_2_S layer being prepared by the cation exchange method,^44^ more precise thickness control of the nanostructured Cu_2_S thin films needs to be optimized to match the short minority carrier diffusion length of the Cu_2_S layer and to achieve higher PEC performance from the nanostructured Cu_2_S photocathode.

To compare the wavelength-dependent photoresponse of Cu_2_S photocathodes based on the nanoplate Cu_2_S thin film and the non-nanostructured Cu_2_S thin film, IPCE measurements were carried out at 0 *V*_RHE_ in the pH 7 electrolyte, and APCEs were calculated based on the IPCE data of Cu_2_S photocathodes and the light absorptance of the corresponding nanoplate Cu_2_S thin film and the non-nanostructured Cu_2_S thin film. As displayed in Figures S13 and 5d, the photocathode based on the nanoplate Cu_2_S thin film shows both higher IPCE value and APCE value than that of the photocathode based on the non-nanostructured Cu_2_S thin film over most of the visible spectrum, particularly in the blue and green regions. Those observations indicate that not only the light absorption but also the charge separation was improved owing to the nanoplate structure, which could also explain the higher photocurrent densities in the *J*–*E* curves. The slightly lower integrated photocurrent density values calculated from the IPCE data of the photocathodes than those in the *J*–*E* curves were probably due to the unaccounted-for photons below 400 nm in the IPCE measurements.

The stability of the photocathode based on the nanoplate Cu_2_S thin film was tested with a chronoamperometry measurement under constant bias at 0 *V*_RHE_ under simulated AM1.5 G illumination (100 mW cm^–2^), showing good stability over at least 6 h (Figure 5e). The initial increase of the photocurrent density is attributed to the activation of the RuO_*x*_ catalyst.^24^ To get the Faradaic efficiency during the stability test, the analysis of the gas product was performed using GC under conditions similar to the stability test. As shown in Figure S14, the Faradaic efficiency was close to 100%. The relatively low efficiency at 10 min, which is called the induction period, was likely due to adhesion of the generated H_2_ and/or unsteady mass transfer, not the headspace of the cell in the initial stage. The promising stability of the Cu_2_S photocathode based on the nanoplate Cu_2_S thin film shows great potential for stable solar hydrogen production based on low-cost Cu_2_S obtained by the solution-processing method, which is favorable for large-area fabrication.

## Conclusions

In summary, phase-pure nanoplate and nanoparticle low chalcocite Cu_2_S thin films were prepared via simple solution processing of CuCl- and CuCl_2_-based molecular inks. The Cu^2+^ cation in the CuCl_2_-based molecular ink was reduced to Cu^+^ by oxidation of the thiol group to the disulfide, enabling the Cu_2_S stoichiometry to be achieved in the resulting films from both inks. The origin of the morphological difference in the final films was not related to the local coordination environment around the copper ions in the molecular inks and was postulated to be due to a difference in the chloride content. After coating with the CdS junction layer, the TiO_2_ protective layer, and the RuO_*x*_ HER catalyst, the FTO/Au/Cu_2_S/CdS/TiO_2_/RuO_*x*_ photocathodes based on the nanoplate and nanoparticle Cu_2_S thin films gave enhanced charge carrier collection toward longer wavelengths compared to the photocathode based on the non-nanostructured Cu_2_S thin film. Especially the photocathode based on the nanoplate Cu_2_S thin film demonstrated a photocurrent density of 3.0 mA cm^–2^ at −0.2 *V*_RHE_ and an onset potential of 0.43 *V*_RHE_ with only 100 nm of nanoplate Cu_2_S layer. Moreover, promising stabilities were observed with almost complete retention in photocurrent after continuous illumination (100 mW cm^–2^) for 6 h. This work provides a simple, cost-effective, and high-throughput method to prepare phase-pure nanostructured Cu_2_S thin films, which will facilitate the development of Cu_2_S photocathodes for scalable and efficient solar hydrogen production.

## Supporting Information Available

SEM images, XPS spectrum, Raman spectra, UV–vis reflectance spectra, Tauc plots, XANES spectra, Fourier transforms, structural parameters, optical images, GIXRD patterns, CV scans, *J*–*E* curves, IPCE spectra, and Faradaic efficiency data (PDF)
