# Supplementary material for: Solution-Processed Cu2S Nanostructures for Solar Hydrogen Production
Source: Chem Mater. 2023 Mar 8;35(6):2371–80. doi: 10.1021/acs.chemmater.2c03489 (PMC10061676; doi:10.1021/acs.chemmater.2c03489)
Supplement: Supplementary file 1 — cm2c03489_si_001.pdf [file cm2c03489_si_001.pdf]

Supporting Information (SI)

Solution Processed Cu_2S Nanostructures for Solar Hydrogen Production

Xi Zhang[a], Stephan Pollitt[b], Gihun Jung[c], Wenzhe Niu[a], Pardis Adams[a], Jan Bühler[a], Nora S. Grundmann[a], Rolf Erni[d], Maarten Nachtegaal[b], Neul Ha[ef], Jisu Jung[ef], Byungha Shin[c], Wooseok Yang[aef]*, S. David Tilley[a]*

[a]*Department of Chemistry, University of Zurich, Winterthurerstrasse 190, 8057 Zurich, Switzerland*

[b]*Paul Scherrer Institut (PSI), Forschungsstrasse 111, 5232 Villigen, Switzerland*

[c]*Department of Materials Science and Engineering, Korea Advanced Institute of Science and Technology (KAIST), 291 Daehak-ro, Yuseong-gu, Daejeon 34141, Republic of Korea*

[d]*Electron Microscopy Center, Empa, Swiss Federal Laboratories for Materials Science and Technology, Überlandstrasse 129, 8600 Dübendorf, Switzerland*

[e]*School of Chemical Engineering, Sungkyunkwan University, 2066 Seobu-ro, Jangan-gu, Suwon-si, Gyeonggi-do 16419, Republic of Korea*

[f]*SKKU Institute of Energy Science and Technology (SIEST), Sungkyunkwan University, Suwon 16419, Republic of Korea*

*Email: wooseok.yang@skku.edu, david.tilley@chem.uzh.ch

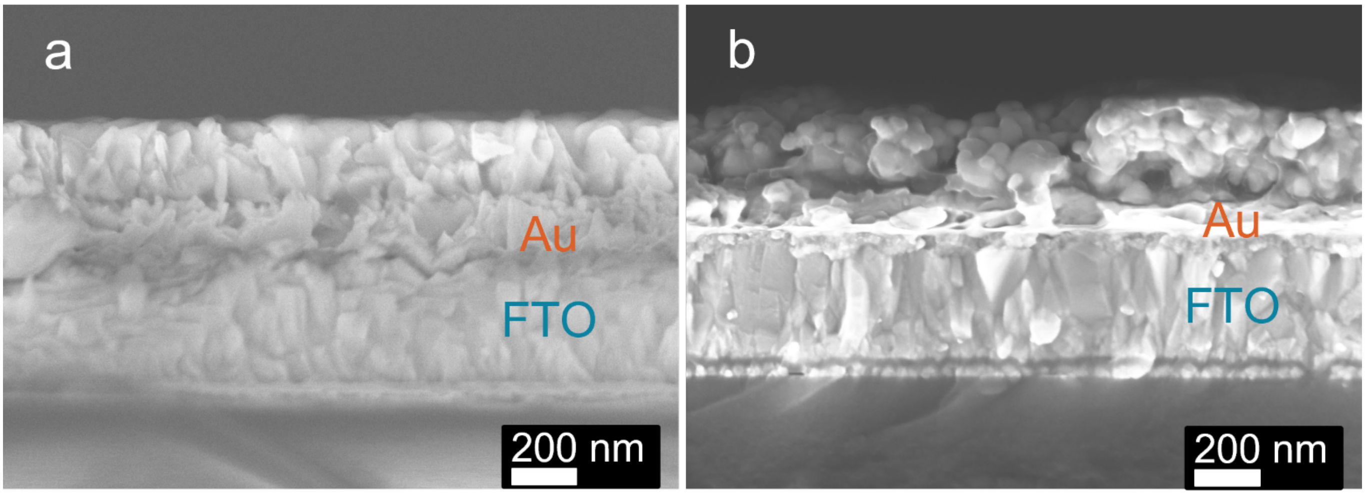

Figure S1. Cross-sectional SEM images of thin films prepared from (a) 0.35 M CuCl ink and (b) 0.35 M $CuCl_2$ ink.

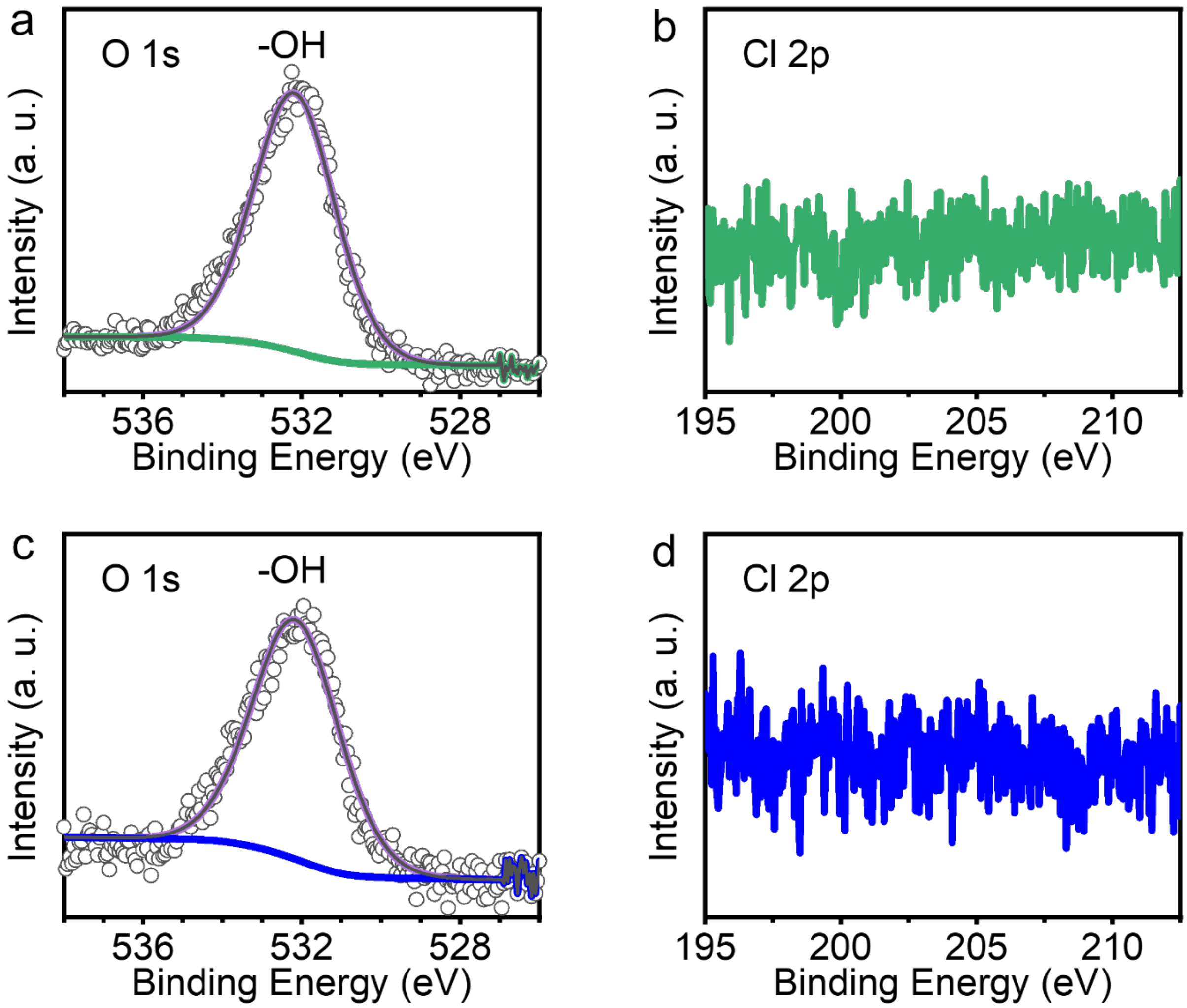

Figure S2. (a, c) O 1s XPS spectrum and (b, d) Cl 2p XPS spectrum of (a, b) nanoplate thin film prepared from 0.35 M CuCl ink and (c, d) nanoparticle thin film prepared from 0.35 M $CuCl_2$ ink.

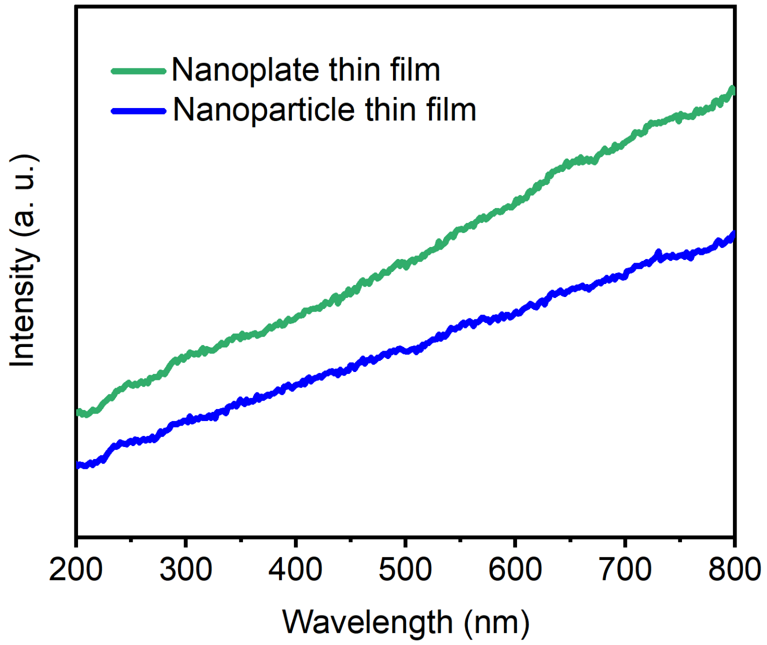

Figure S3. Raman spectra of prepared nanoplate and nanoparticle thin films.

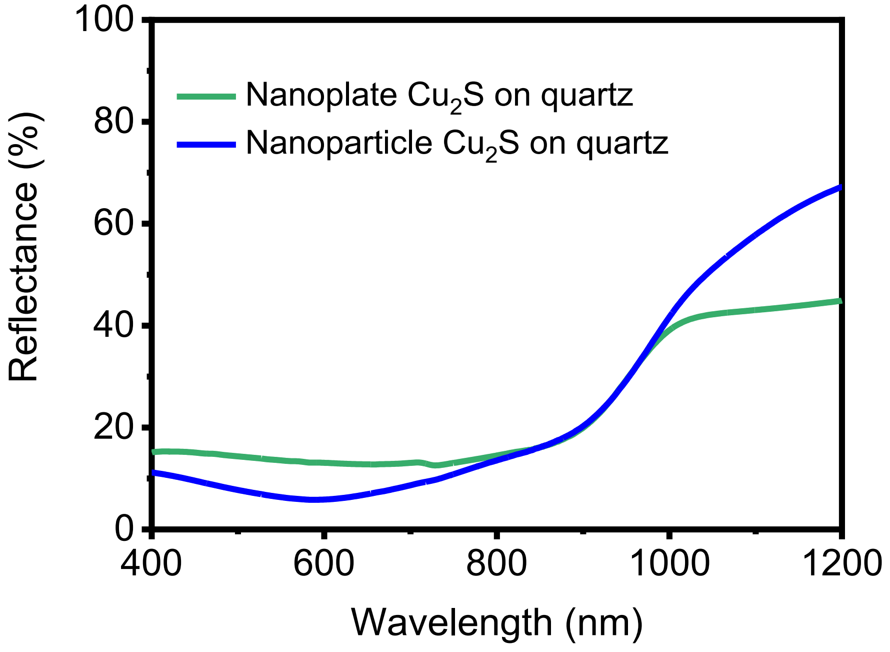

Figure S4. The reflectance spectra of nanoplate and nanoparticle Cu_2S thin films on quartz substrates.

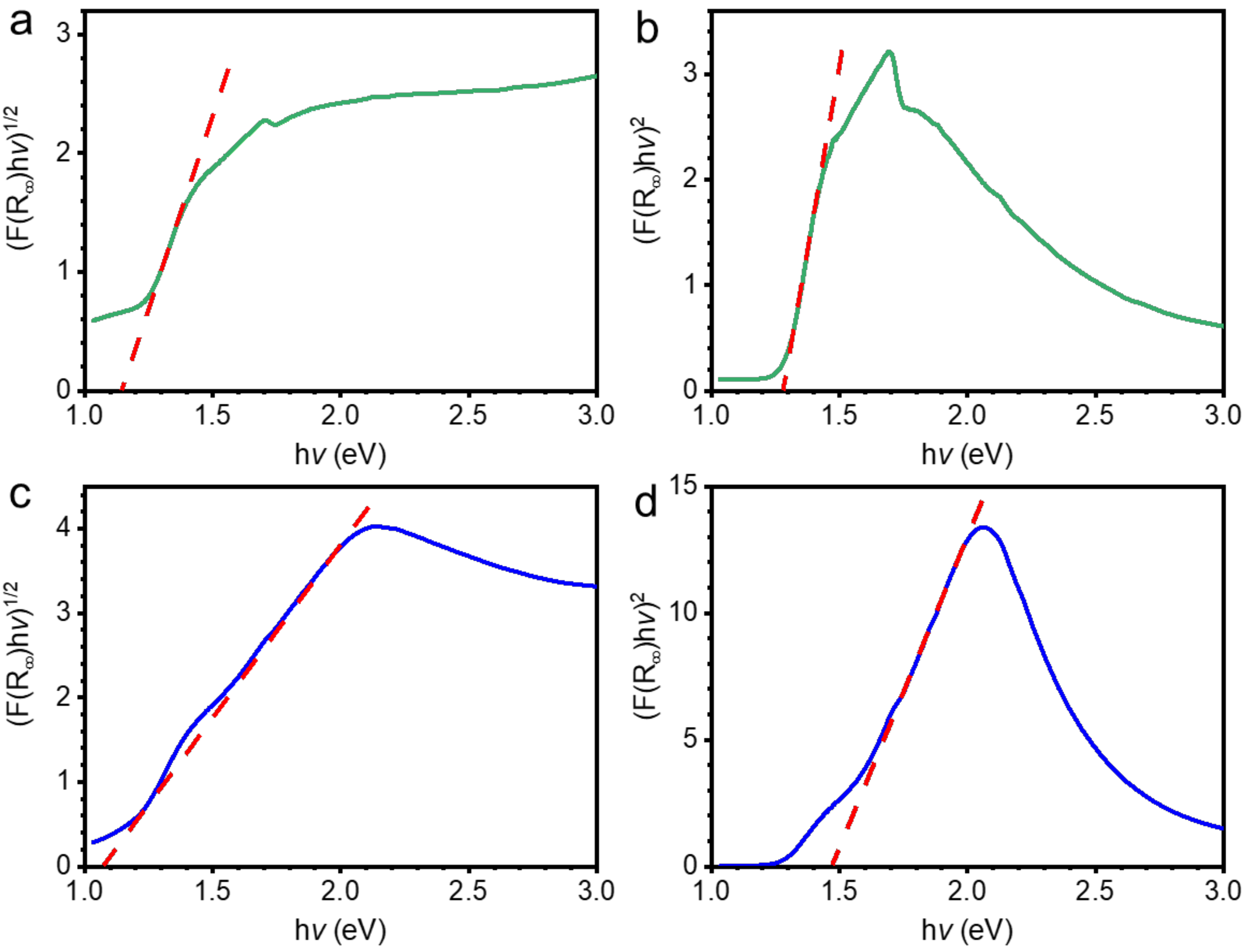

Figure S5. Tauc plots of nanoplate (a, b) and nanoparticle (c, d) Cu_2S thin films to determine their indirect (a, c) and direct (b, d) band gap energies (Eg). The linear parts of the plots are extrapolated to the x-axis.

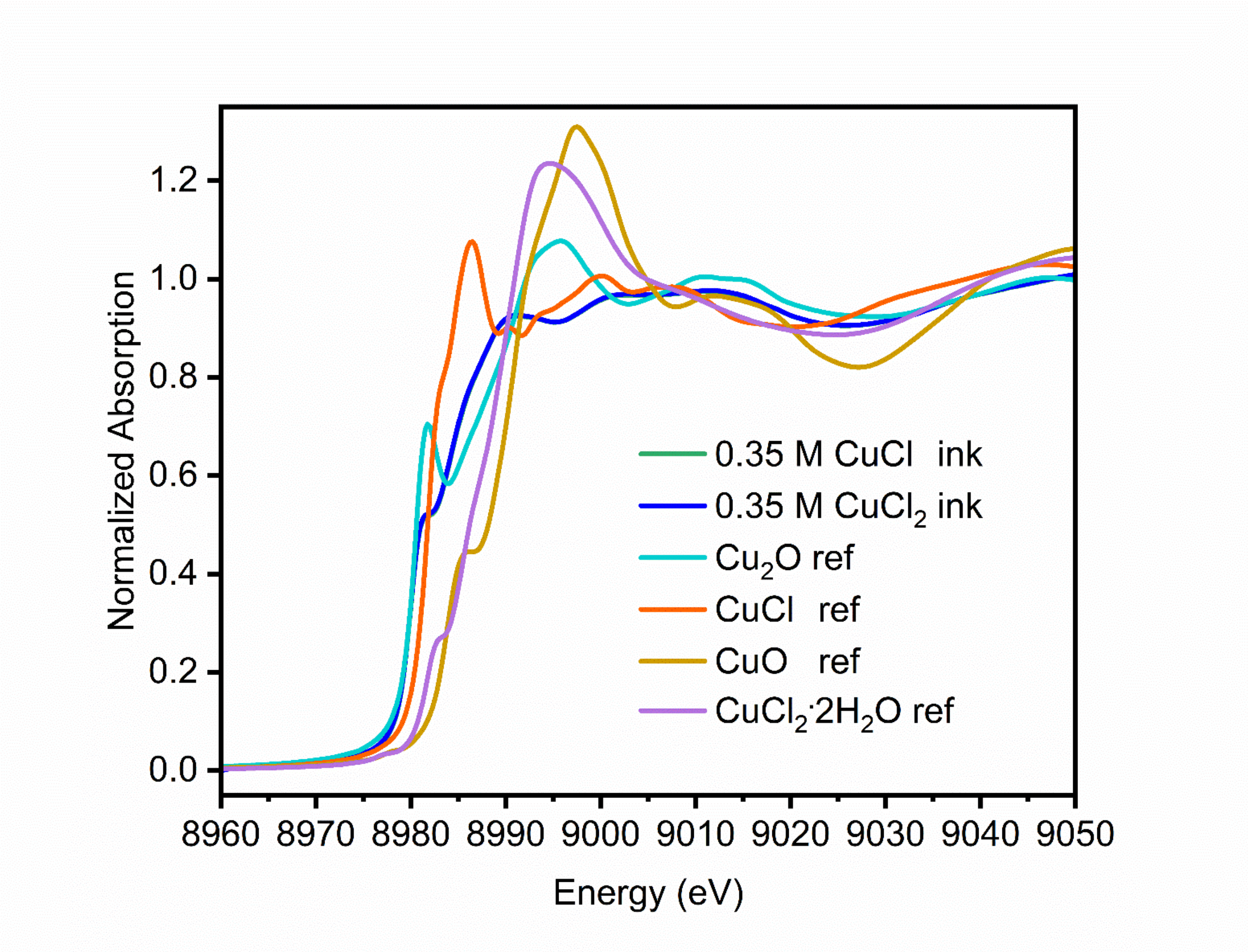

Figure S6. Cu K-edge X-ray absorption near-edge structure (XANES) spectra for 0.35 M CuCl and $CuCl_2$ molecular inks (which overlap fully) along with pressed Cu_2O, CuO, CuCl and $CuCl_2{\cdot}2H_2O$ powder references.

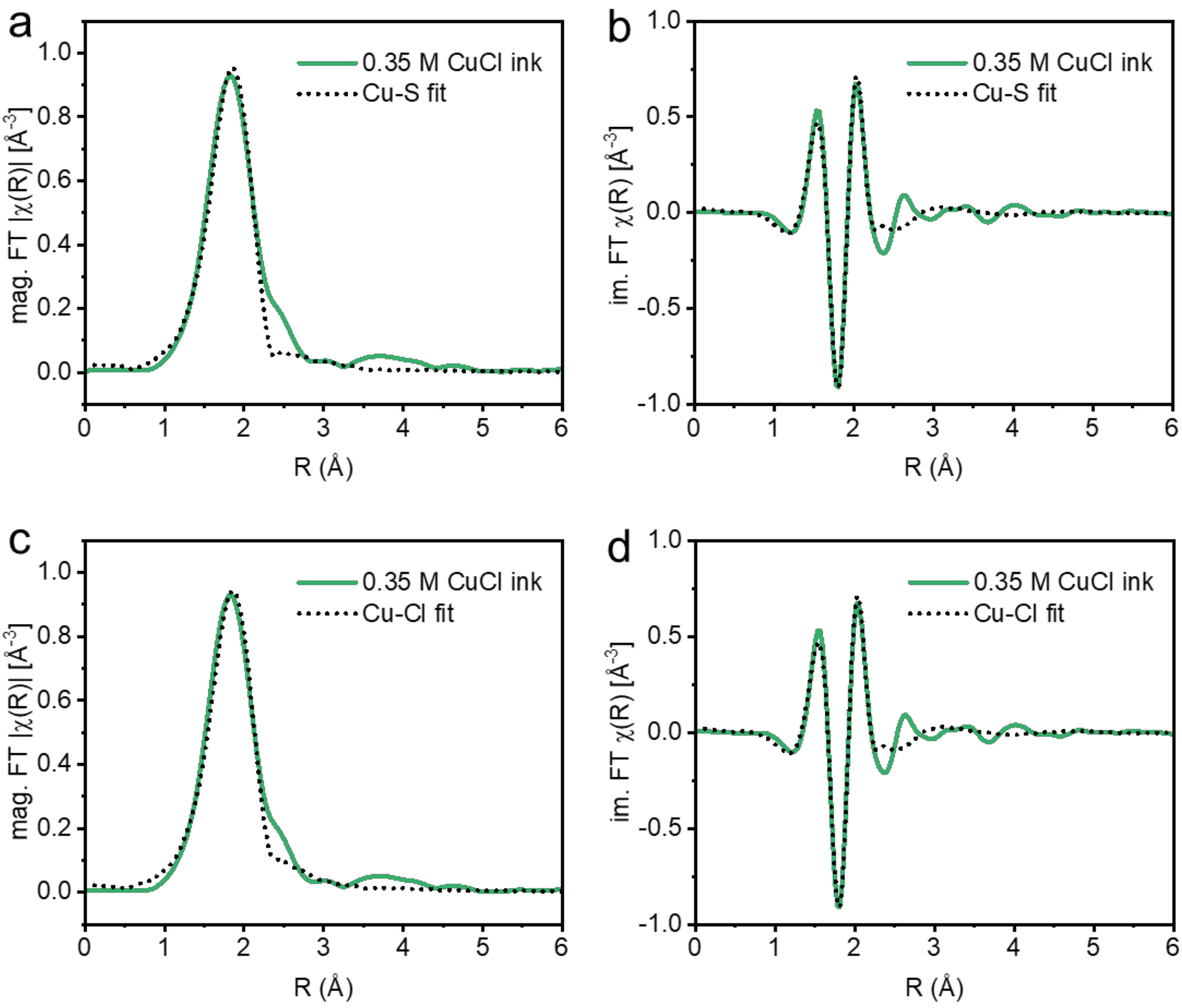

Figure S7. The magnitude (a, c) and imaginary part (b, d) of the Fourier transforms (FT) of Cu K-edge k^3-weighted extended X-ray absorption fine structure (EXAFS) spectra of 0.35 M CuCl ink. Green solid lines represent experimental data and black dash line represents fits based on chalcocite (Cu_2S) and nantokite (CuCl).[1,2]

Table S1. Structural parameters of 0.35 M CuCl ink obtained from Cu-S fit based on chalcocite (Cu_2S) and Cu-Cl fit based on nantokite (CuCl).[1,2]

Fit	CN	R (Å)	σ^2 (Å^2)	E_0 (eV)
Cu-S fit	2.5 (+/- 0.4)	2.28 (+/- 0.01)	0.008 (+/- 0.002)	4.4 (+/- 1.5)
Cu-Cl fit	2.2 (+/- 0.3)	2.26 (+/- 0.01)	0.008 (+/- 0.002)	4.9 (+/- 1.3)

[a]CN: coordination number, R: interatomic distance, σ^2: pseudo Debye-Waller factor, E_0: shift in threshold energy, S_0^2: intrinsic loss factor fixed to 0.8 determined from fitting a Cu metal foil spectrum.

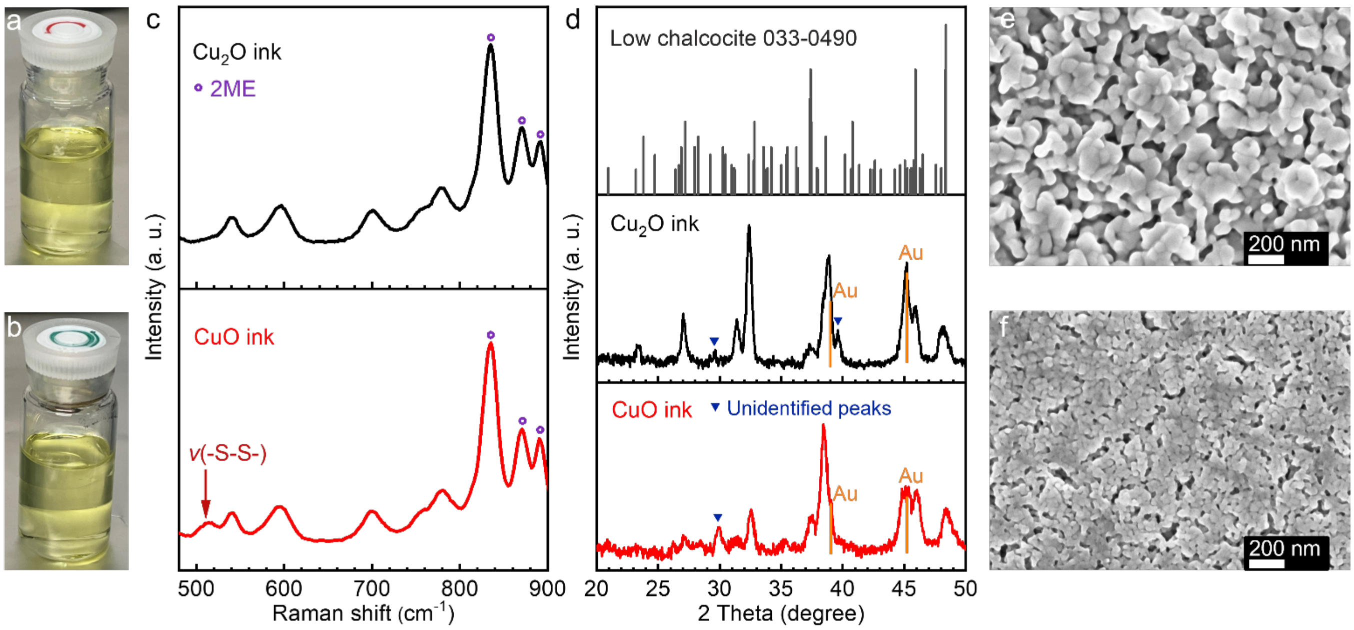

Figure S8. (a) Optical image of 0.175 M Cu_2O ink. (b) Optical image of 0.35 M CuO ink. (c) Molecular structure analysis of 0.175 M Cu_2O and 0.35 M CuO ink molecular inks by liquid-phase Raman spectroscopy normalized according to the 2ME peaks. (d) GIXRD patterns of thin films prepared from 0.175 M Cu_2O ink and 0.35 M CuO ink. The unidentified peaks do not match with any copper oxides or copper chlorides. (e) Plan view SEM image of the thin film prepared from the 0.175 M Cu_2O ink. (f) Plan view SEM image of the thin film prepared from the 0.35 M CuO ink.

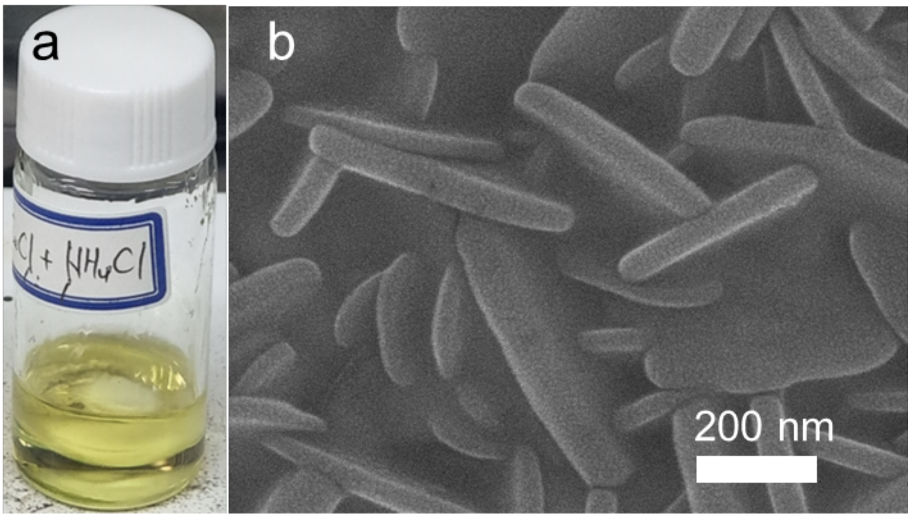

Figure S9. (a) Optical image of the (0.35 M CuCl+0.35 M NH_4Cl) ink. (b) Plan view SEM image of the thin film prepared from the (0.35 M CuCl+0.35 M NH_4Cl) ink.

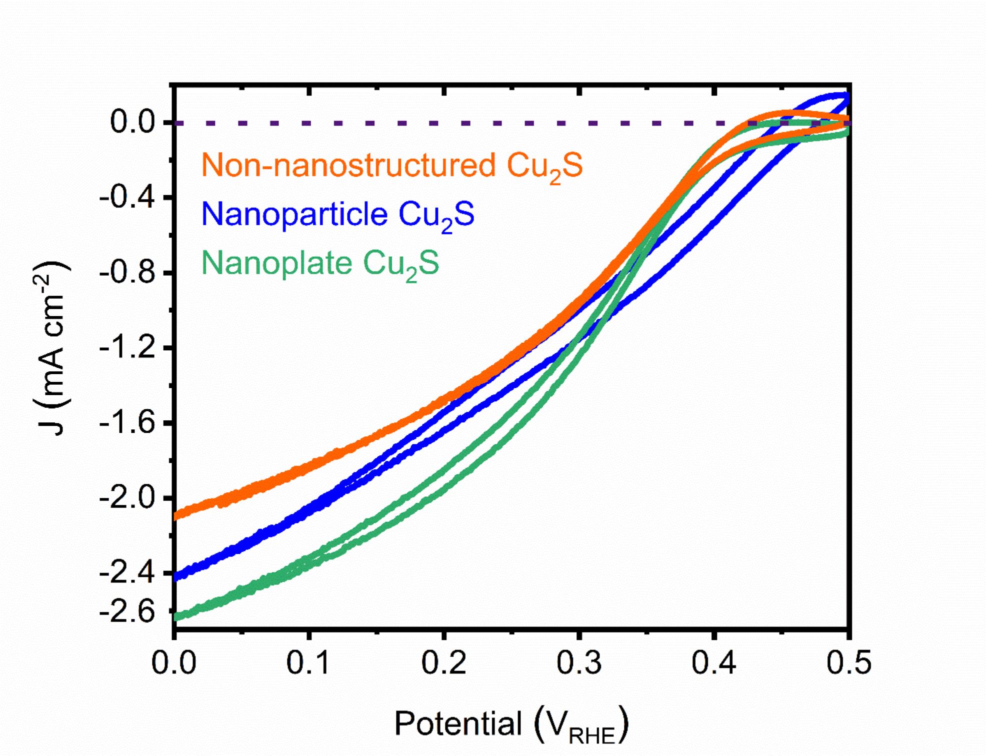

Figure S10. Cyclic voltammetry (CV) scans of Cu_2S photocathodes prepared from nanoplate, nanoparticle, and non-nanostructured Cu_2S thin films. Note: the non-nanostructured Cu_2S data comes from our previous work.[3]

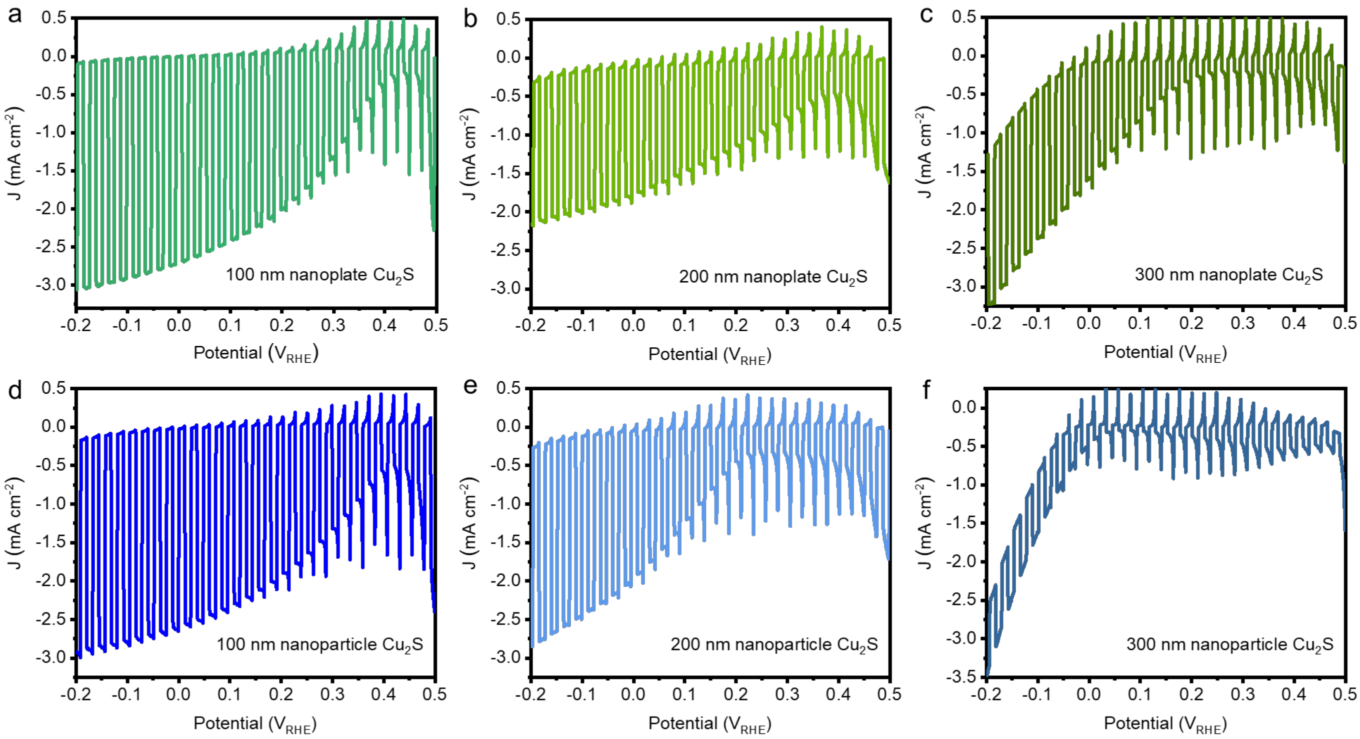

Figure S11. *J-E* curves of Cu_2S photocathodes based on different thickness of nanostructured Cu_2S thin films under simulated on-off AM1.5 G illumination (100 mW cm^{-2}).

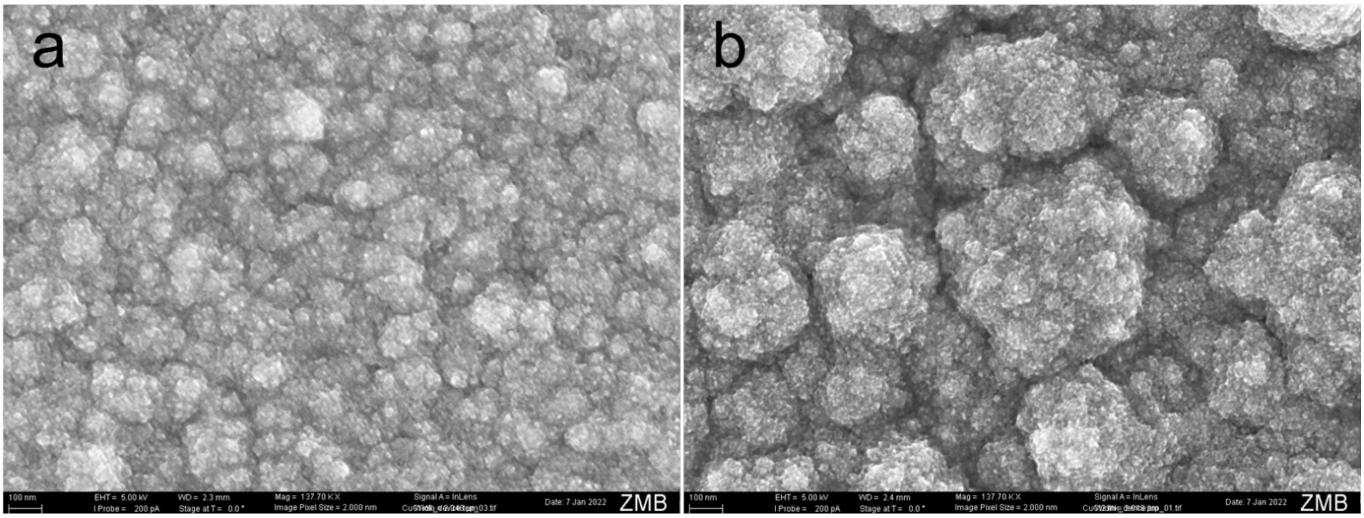

Figure S12. The top-view SEM images of Cu_2S photocathodes (FTO/Au/ Cu_2S/CdS/TiO_2/RuO_x) based on (a) nanoplate Cu_2S thin film and (b) nanoparticle Cu_2S thin film.

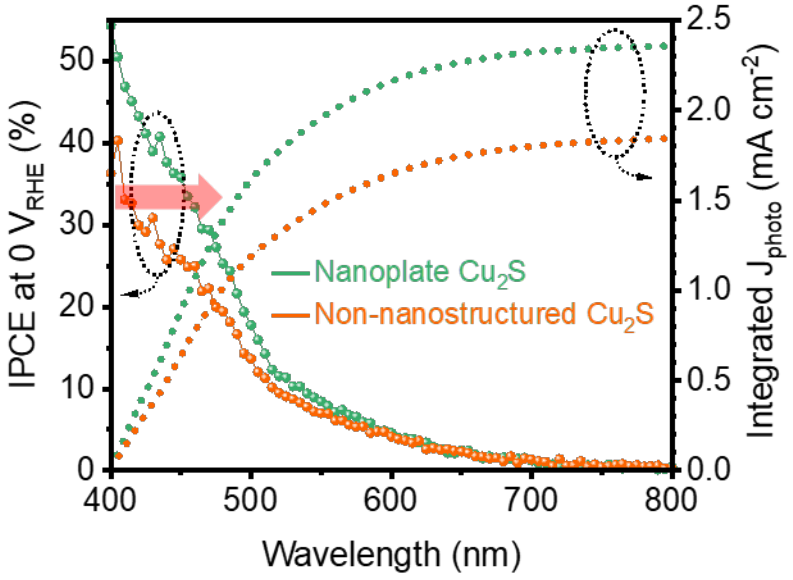

Figure S13. Incident photon-to-current efficiency (IPCE) spectra of the Cu_2S photocathodes at an applied bias of 0 V_{RHE} under monochromatic illumination with 10% white light bias.

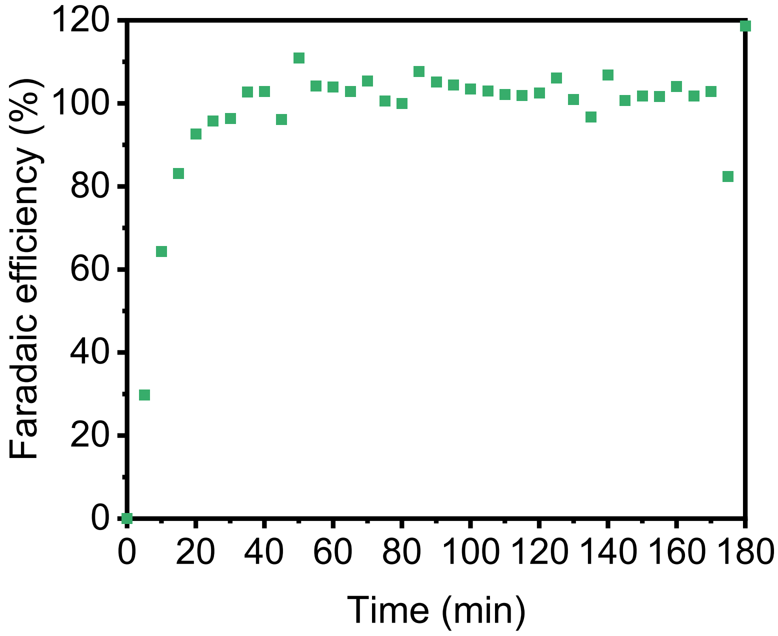

Figure S14. Faradaic efficiency of the Cu_2S photocathode based on the nanoplate Cu_2S thin film under constant bias at 0 V_{RHE} performed in a 1.0 M phosphate buffer solution (pH 7.0) using an LED light source where the intensity was adjusted to obtain similar current densities as those obtained under simulated AM1.5 G illumination (100 mW cm^{-2}).

References:

1 H. T. Evans, *Nature Physical Science*, 1971, **232**, 69–70.

2 R. W. G. Wyckoff and E. Posnjak, *J. Am. Chem. Soc.*, 1922, **44**, 30–36.

3 X. Zhang, W. Yang, W. Niu, P. Adams, S. Siol, Z. Wang and S. D. Tilley, *ChemSusChem*, 2021, **14**, 3967–3974.
